# 带蒂心包脂肪垫包裹支气管吻合口预防支气管吻合口瘘的临床效果观察

**DOI:** 10.3779/j.issn.1009-3419.2020.104.01

**Published:** 2020-05-20

**Authors:** 小云 黎, 汉宇 邓, 希 郑, 大兴 朱, 清华 周, 小军 唐

**Affiliations:** 1 646000 泸州，西南医科大学 Southwest Medical University, Luzhou 646000, China; 2 611135 成都，四川大学华西医院肺癌中心 Lung Cancer Center, West China Hospital, Sichuan University, Chengdu 611135, China

**Keywords:** 中心型肺癌, 支气管袖式肺叶切除, 带蒂胸膜脂肪带, 支气管胸膜瘘, Central lung cancer, Bronchial seeve lobectomy, Pedicled pericardial fat flap, Bonchial pleural fistula

## Abstract

**背景与目的:**

支气管袖式肺叶切除是中心型肺癌重要的手术方式，它是最能体现“最大程度切除肿瘤，同时最大程度保留肺功能”的肺癌手术原则。支气管胸膜瘘是支气管袖式肺叶切除最严重的手术并发症，严重威胁患者的生命安全。本文将总结带蒂心包脂肪垫包裹支气管吻合口在预防支气管袖式肺叶切除术后支气管吻合口瘘的临床效果。

**方法:**

回顾性分析四川大学华西医院肺癌中心2016年1月-2019年5月期间行支气管袖式肺叶切除术，并用带蒂心包脂肪垫包裹支气管吻合口的39例中心型肺癌患者临床资料，观察该组患者术后并发症，尤其是支气管吻合口相关并发症发生情况。

**结果:**

该组患者手术后恢复良好，均于术后6 d-14 d内出院；30 d内无支气管胸膜瘘发生，无因胸腔内出血再次行手术病例，无严重心律失常，无严重肺部感染及呼吸衰竭发生；术后继续随访期间，术后6个月发生重度吻合口狭窄导致术侧残余肺不张1例。

**结论:**

支气管袖式肺叶切除的肺癌患者，术中用带蒂心包脂肪垫包裹支气管吻合口，可有效预防术后吻合口瘘相关并发症的发生，从而提高手术安全性。

## Abstract (en)

**Background and objective:**

Bronchial sleeve lobectomy is essential surgical approach to treat centralized lung cancer. It is the best reflected the principle of lung cancer surgery, "remove tumor completely while minimize pulmonary function loss". Bronchial pleural fistula (BPF) is not common but very severe complication of bronchial sleeve lobectomy, that is usually fatal. Present article is to explore clinical effect on prevention of bronchial pleural fistula (BPF) in bronchial sleeve lobectomy, by wrapping brachial anastomosis with pedicled pericardial fat flap.

**Methods:**

Clinical data of 39 non-small cell lung cancer (NSCLC) patients who underwent surgical resection during January 2016 to May 2019 in Lung Cancer Center of West China Hospital, Sichuan University were collected and retrospectively analyzed. All of the patients underwent bronchial sleeve lobectomy and a brachial anastomosis wrapping with pedicled pericardial fat flap.

**Results:**

All patients recovered well and were discharged within 6 d-14 d after operation. No BPF occurred, nor other severe complications, such as reoperation needing intrathoracic bleeding, several pneumonia and respiratory failure, and life threatening cardiac arrhythmia. Only one patient (1/39) had several anastomotic stenosis and consequential atelectasis of residual lung in operative side 6 months after surgery.

**Conclusion:**

Wrapping bronchial anastomosis with pedicled pericardial fat flap in bronchial lobectomy for centralized NSCLC is a simple and effective approach to prevent BPF, thus safety of the operation could be significantly improved.

在中心型非小细胞肺癌(non-small cell lung cancer, NSCLC)的外科治疗中，支气管袖式肺叶切除术(bronchial sleeve lobectomy, BSL)可以实现与全肺切除相似的彻底性，同时能保留更多的正常肺组织和肺功能^[1]^。因此，BSL逐渐成为中心型NSCLC外科治疗的优选手术方式。来自于欧洲和美国的多项大样本研究结果显示，无论是术后30 d还是90 d的死亡率和严重并发症发生率，支气管袖式肺叶切除均低于全肺切除患者，术后长期生活质量也显著优于全肺切除患者^[2-4]^。基于上述研究结果，美国胸外科医师协会(American College of Chest Physicians)推荐支气管袖式肺叶切除作为中心型NSCLC外科治疗的优选手术方式^[5]^。相对于全肺切除而言，支气管袖式肺叶切除手术更复杂，对手术医生技术要求更高。另外，虽然支气管袖式肺叶切除手术本身已经相当成熟，但其术后并发症发生率仍较高。支气管吻合口瘘是支气管袖式肺叶切除术后最严重的并发症之一，一旦发生，可能导致严重而持续的胸腔感染，感染时可能累及瘘口临近重要组织，如肺动脉等，导致致命性大出血^[6]^。吻合口瘘即使经过治疗得以愈合，后期可发生支气管瘢痕和肉芽过度生长导致严重支气管狭窄。降低支气管袖式肺叶切除术后支气管胸膜瘘的发生，可以提高该手术的安全性及患者的远期效果。有多项研究^[7, 8]^报道，用人工材料或患者自身组织对支气管吻合口进行覆盖和包裹，有可能降低支气管胸膜瘘的发生。但对于支气管吻合口是否需要包裹以及用何种材料包裹，目前仍有争议。因此本研究拟采用回顾性分析，对中心型NSCLC患者行支气管袖式肺叶切除时，用带蒂心包脂肪垫包裹支气管袖式吻合口的近期疗效进行总结，旨在探索一种简易可行、能降低支气管袖式肺叶切除术后支气管胸膜瘘的方法。

## 1 资料与方法

### 1.1 临床资料

收集2016年1月-2019年5月期间在四川大学华西医院肺癌中心接受支气管袖式肺叶切除术的39例中心型NSCLC患者的临床资料，并对这些临床资料进行回顾性总结、分析。纳入本研究的所有患者均由同一个医疗组完成，患者一般资料见表 1。其中男性38例、女性1例，年龄43-74 (59.6±8.4)岁；肿瘤位于右肺上叶14例，右肺中叶1例，右肺下叶2例，同时累及右肺中、下叶2例，左肺上叶14例，左肺下叶6例；术后病理证实为鳞癌32例，腺癌3例，肉瘤样癌+鳞癌4例；合并高血压病患者4例，冠心病2例，糖尿病1例，同时合并高血压和糖尿病1例，肝硬化代偿期1例；术前行新辅助化疗1例。所有患者术前均经纤支镜检查发现肿瘤累及病变肺叶支气管开口，并接受胸部增强计算机断层扫描(computed tomography, CT)、全腹增强CT、头颅增强磁共振成像(magnetic resonance imaging, MRI)、骨扫描，评估肿瘤侵犯范围并排除远处转移；同时完善肺功能、心脏超声、心电图等常规术前检查，排除心、肺、肝等重要脏器功能明显异常等手术禁忌证。

**1:** 39例患者的一般资料 General characteristics of 39 patients

Parameter	Data
Age (yr)	43-74 (59.6 ± 8.4)
Gender	
Female	38 (97.4%)
Male	1 (2.6%)
Lesion location	
Right upper lobe	14 (35.9%)
Right middle lobe	1 (2.6%)
Right lower lobe	2 (5.1%)
RML and RLL	2 (5.1%)
Upper left lobe	14 (35.9%)
Left lower lobe	6 (15.4%)
Preoperative comorbidities	
Hypertension	4 (10. 3%)
Diabetes	1 (2.6%)
Coronary heart disease	2 (5.1%)
Hypertension with diabetes	1 (2.6%)
Cirrhosis	1 (2.6%)
Postoperative pathological type	
Squamous cell carcinoma	32 (82.1%)
Adenocarcinoma	3 (7.7%)
Sarcomatoid carcinoma with squamous Carcinoma	4 (10.2%)

### 1.2 手术方法与技巧

所有患者均采用开胸手术，行支气管袖式肺叶切除及系统性淋巴结清扫。主要手术步骤如下：静吸复合全麻、双腔管气管插管，侧卧位施术；作后外侧剖胸切口，拟行上叶切除的患者沿第5肋骨上缘入胸，拟行中、下叶切除者沿第6肋骨上缘入胸；常规处理肺血管；游离支气管，分别切断保留肺叶支气管起始部及主支气管远端，使支气管切缘距肿瘤距离超过0.5 cm以上，支气管两端切缘均送术中冰冻切片检查，排除切缘癌残留；清扫肺门、隆突下及纵隔淋巴结；用3-0"薇乔"可吸收线间断缝合方法作主支气管-叶支气管端端吻合；无菌生理盐水冲洗胸腔，请麻醉师鼓肺，检查支气管吻合口无漏气后，用电刀自前肋膈角处向上游离心包外脂肪垫，作成带蒂胸膜脂肪带，360°包裹支气管吻合口，使胸膜脂肪带的粗糙面接触支气管吻合口，并缝合2针-3针，将心包脂肪带固定在支气管吻合口前壁，防止其术后移位(图 1，图 2)。

**1:**
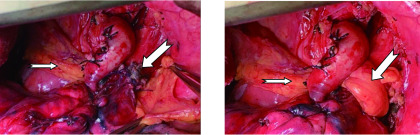
术中用带蒂心包脂肪带包裹支气管吻合口的方法。小箭头显示带蒂心包脂肪带的近端部分，大箭头显示包裹前和包裹后的支气管吻合口 Wrapping bronchial anastomosis with pedicled pericardial fat flap. Small arrow showing proximal part of pedicled pericardial fat flap; big arrow showing unwrapped or wrapped bronchial anastomosis

**2:**
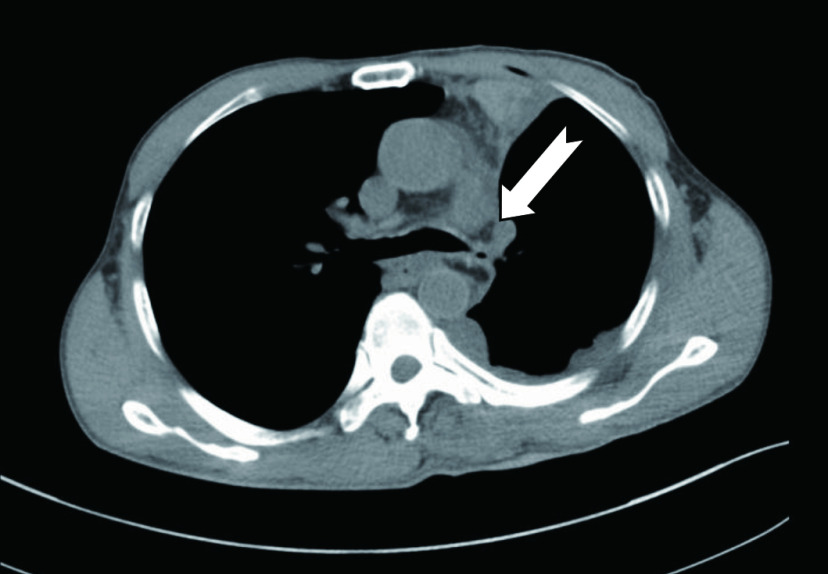
术后支气管吻合口及其周围的带蒂心包脂肪带的CT影像。白色箭头显示的脂肪密度组织为包裹在支气管吻合口周围的带蒂心包脂肪带 CT image of bronchial anastomosis and pedicled pericardial fat flap around it. White arrow showing pedicled pericardial fat flap wrapping bronchial anastomosis. CT: computed tomography

### 1.3 术后治疗

患者术后送重症监护室(intensive care unit, ICU)监护治疗，拔除气管导管前用纤支镜观察吻合口情况，清除支气管腔内，尤其是支气管吻合口附近的血凝块和分泌物。术后使用抗生素3 d-5 d预防感染；术后每日静脉使用甲强龙40 mg-80 mg，持续2 d-3 d，减轻吻合口水肿；其他呼吸道治疗、护理及对症治疗，如雾化吸入、止痛治疗等与常规胸科手术相同。对于术后痰多，自主咳嗽排痰困难者，每日给予纤支镜吸痰1次-2次，直至能自主有效咳嗽排痰。患者病情平稳后转普通病房治疗。术后2 d-3 d，如果胸腔引流液小于200 mL/d，而且无持续性漏气，复查胸片示肺复张良好，则可拔除胸腔引流管；鼓励患者积极咳嗽排痰，尽早下床适当活动。根据术后病理检查结果决定术后随访及辅助治疗(化疗、放疗、靶向治疗、免疫治疗等)方案。视患者恢复情况，术后6 d-14 d出院。

### 1.4 随访方法

出院时嘱患者术后30 d回院复查胸部CT，了解患者手术恢复情况，及有无胸腔积液、积气及肺部感染、不张等情况，并根据患者术后病理检查结果，与肿瘤科医生一起为患者制定术后辅助治疗或定期复查计划。术后3个月，再次复查胸部增强CT，如CT显示术侧肺不张或明显支气管狭窄，安排纤维支气管镜检查，了解有无支气管及吻合口狭窄、肿瘤复发等情况，并视情况不同采用相应治疗措施。

### 1.5 观察指标

主要观察患者术后吻合口瘘及吻合口狭窄发生率，同时收集患者手术时间、术中失血量、术后ICU留置时间及术后住院时间、术后抗生素使用时间、术后其他主要并发症发生率。

### 1.6 统计学方法

采用统计学软件SPSS 20.0进行分析。计数资料采用率(%)表示，计量资料采用均数±标准差(Mean±SD)表示。

## 2 结果

所有患者均顺利完成手术，无术中大出血及术后30 d内死亡病例。围术期指标见表 2。术中出血量约100-800 (206.9±162.4) mL，手术时间125-258 (173.5± 31.3) min，其中手术时间3 h以内27例(69.2%)，超过3 h 12例(30.8%)主要原因是：胸腔致密黏连、肺门淋巴结肿大或钙化与肺门结构黏连致密、病灶侵及肺血管、支气管、心包等邻近组织，ICU住院时间1-4 (1.7±0.9) d，术后抗生素使用时间3-5 (3.9±0.7) d，术后住院时间6-14 (8.2±2.1) d。术后并发症发生率20.5% (8/39)，其中肺部感染2例(5.1%)，房颤3例(7.7%)，切口感染2例(5.1%)，吻合口狭窄1例(2.6%)。所有患者均顺利出院，术后随访4个月-31个月，1例支气管袖式左肺下叶切除患者在术后6个月发生重度吻合口狭窄及左肺上叶不张，经纤支镜检查显示为吻合口瘢痕性狭窄，无吻合口复发病例。

**2:** 39例患者的围术期指标 Perioperative parameters of 39 patients

Parameters	Data
Postoperative complications	
Anastomotic fistula	0 (0.0%)
Narrow anastomosis	1 (2.6%)
lung infection	2 (5.1%)
Atrial fibrillation	3 (7.7%)
Incision infection	2 (5.1%)
Operation time (min)	125-258 (173.5 ± 31.3)
Within 3 hours	27 (69.2%)
More than 3 hours	12 (3.8%)
Intraoperative blood loss (mL)	100-800 (106.9 ± 162.4)
Length of stay in ICU (d)	1.4 (1.7% ± 0.9%)
Postoperative hospital stay (d)	6-14 (8.2% ± 2.1%)
Antibiotic use time (d)	3-5 (3.9% ± 0.7%)

## 3 讨论

相对单纯肺叶切除而言，支气管袖式肺叶切除手术更复杂，并发症风险更高。文献^[9]^报道，吻合口瘘和狭窄等支气管吻合口相关并发症发生率高达10%，其中支气管吻合口瘘是最严重的术后早期并发症，严重威胁患者的生命安全，同时也会延误患者术后辅助治疗。支气管袖式肺叶切除术后发生支气管吻合口瘘的原因主要有：①支气管吻合技术不恰当；②支气管吻合口血供不良；③吻合口发生感染；④术前接受放射治疗或其他合并症，如糖尿病等导致组织愈合能力低下^[10]^。因此，降低支气管吻合口瘘的发生，应该针对上述原因采取相应措施。在本组患者中，我们在支气管吻合前，充分游离残余肺叶的血管和肺门结构，防止支气管吻合口张力过高，并在吻合过程中防止缝合过密或过稀；术后根据患者具体情况，采用纤支镜吸痰等措施及时、有效清除患者呼吸道分泌物，防止支气管吻合口感染；积极治疗合并症，及时纠正缺氧、贫血、低蛋白血症，有效控制血糖等，提高组织愈合能力。

有文献报道，采用自身材料或人工材料对支气管吻合口进行覆盖和包裹，有可能进一步降低支气管袖式切除术后支气管吻合口瘘的发生。Yildizeli等^[11]^用胸膜或心包瓣包裹支气管吻合口，其吻合口瘘发生率为2.3%。Rendina等^[12]^用肋间肌瓣包裹支气管吻合口，并发症发生率为2.7%。Matsuoka等^[13]^回顾性分析了37例采用游离心包脂肪包裹支气管残端的患者，术后均未发生支气管胸膜瘘。Ichinose等^[14]^用动物实验方法研究了心包脂肪垫覆盖防止支气管胸膜瘘的作用机制，发现心包脂肪组织能诱导新生血管的形成并产生与组织修复相关的细胞因子[白介素1 (interleukin 1, IL-1)、肿瘤坏死因子(tumor necrosis factor, TNF)和IL-6等]，且其能力强于大网膜、肋间肌等，从而有利于防止肺叶切除术后支气管胸膜瘘的发生。本研究中，作者在支气管袖式肺叶切除术中，利用患者术侧心包外脂肪垫，制作了带蒂的脂肪带，360°包裹支气管吻合口，以防止支气管吻合口瘘的发生。结果显示，39例支气管袖式肺叶切除患者，术后30天内无一例发生支气管吻合口瘘。进一步随访发现，只有1例患者在术后6个月发生术侧残余肺叶不张，纤支镜检查证实为支气管吻合口重度瘢痕形成导致支气管闭塞。作者推测，带蒂心包脂肪带包裹，预防支气管吻合口瘘的机制可能有以下几点：①改善支气管吻合口附近血供，提高吻合口愈合能力；②如果吻合口发生小的瘘口，带蒂脂肪垫的包裹可防止呼吸道分泌物扩散到胸膜腔，从而降低了感染的范围和强度，有利于瘘口的自身修复、愈合。

本研究结果显示，带蒂心包外脂肪垫包裹支气管袖式吻合口可能有助于减少术后支气管吻合口瘘的发生，为临床提供了一种选择。当然，本研究为单中心回顾性分析，缺乏对照且病例数较少，还有待于在今后工作中积累更多经验，来进一步证实该方法的近期和远期效果。
